# Congenital Dyserythropoietic Anemia Type II With Myelofibrosis in an Adult Patient: A Report of a Rare Case With a Brief Review

**DOI:** 10.7759/cureus.58515

**Published:** 2024-04-18

**Authors:** Shruti Shemawat, Shweta Bansal, Arpita Mathur, Anjana Mittal, Manoj Sharma

**Affiliations:** 1 Pathology, Mahatma Gandhi University of Medical Sciences & Technology, Jaipur, IND

**Keywords:** sec23b, hemolytic anemia, myelofibrosis, splenomegaly, congenital dyserythropoietic anaemia

## Abstract

Congenital dyserythropoietic anemias (CDAs) are rare hereditary disorders, of which type II CDA is the most common. Mutations in the *SEC23B *gene located on chromosome 20 result in this autosomal recessive disorder. In this case report, we present a case of CDA II with unique biopsy findings being detected via genetic testing. A female aged 30 years presented with major complaints of pallor weakness and easy fatiguability since childhood. The patient gave a history of 25 units of blood transfusion, the majority of which were transfused during pregnancy, followed by regular transfusions thereafter. On examination, all her vitals were in the normal range. Pallor, frontal bossing, and malocclusion of teeth were noted. Her laboratory workup showed the following: hemoglobin (Hb): 3.7 g/dl; mean corpuscular volume: 83 fl; mean corpuscular Hb: 29 g/dl; mean corpuscular Hb concentration: 34.9 g/dl; red cell distribution width: 30.4%; reticulocyte count (RC): 6.2%; corrected RC: 1.3%; lactate dehydrogenase: 441 IU/L; direct Coombs test/indirect Coombs test: negative; serum iron: 242 microgram/dl; transferrin saturation: 96.08%; ferritin: 1,880 ng/ml; and normal high-performance liquid chromatography and eosin-5'-maleimide binding test. The peripheral blood film showed normocytic normochromic anemia with anisopoikilocytosis in the form of a few spherocytes. No immature cells were seen. After obtaining the patient’s consent, we performed a hereditary hemolytic anemia gene analysis test, which showed homozygous missense variation in exon 12 of the *SEC23B* gene. The bone marrow examination showed hyperplasia in the erythroid series with dyserythropoiesis, and surprisingly, myelofibrosis grade I-II (WHO 2017) was also observed on biopsy. Patients with CDA type II generally present with variable degrees of anemia along with pallor, icterus, splenomegaly, gallstones, and iron overload. In our case, the diagnosis of CDA type II was made at an adult age. Also, evidence of myelofibrosis was noted in our case, making it worth reporting. The use of a hereditary hemolytic anemia gene analysis panel test came as a rescue for its exact diagnosis. This case report emphasizes the role of molecular genetic testing for early and accurate diagnosis, which, in turn, could help in appropriate treatment planning and proper genetic counseling. The prevalence of CDA type II is still vaguely known; hence, extensive workup of persistent anemias and proper follow-up would be beneficial.

## Introduction

Congenital dyserythropoietic disorders are a group of rare hereditary diseases described initially in 1951 by Wolff and Von Hofe [1]. Further subcategorization of congenital dyserythropoietic anemia (CDA) into types I, II, and III was outlined by Heimpel and Wendt in 1968 [2]. Currently, a total of six subtypes have been identified, of which type II CDA, inherited as an autosomal recessive trait, is the most common subtype [3]. CDA type II is mostly seen in Mediterranean and European populations; however, sporadic cases have been reported from India and nearby countries [4].

In CDA type II, there is abnormal processing of n-glycans, leading to defects in glycosylation of the proteins that are present in the red cell membrane, namely band 4.5 (glucose transporter 1) and band 3 (anion exchange protein transporter 1). Initially, the pathogenesis of CDA type II was suspected to be due to abnormal Golgi enzymes (N-acetylglucosaminyltransferase II and α-mannosidase II). In 2009, it was discovered that the biochemical hallmark of defective glycosylation occurred due to a mutation in the *SEC23B* gene mapped to chromosome 20. The *SEC23B *mutation resulted in the defective assembly of coat protein complex (COP) II components. Clinically, the patients present with mild to severe symptoms ranging from anemia, jaundice, and sometimes iron overload and hydrops fetalis in severe cases [5]. Due to their rare occurrence as well as their overlapping clinical and morphological blood picture, CDAs can often be misdiagnosed as hemolytic anemias. This case report unveils the unusual clinical presentation and biopsy findings of CDA type II and emphasizes the role of genetic testing in solving such clinical quandaries.

## Case presentation

**Figure 1:**
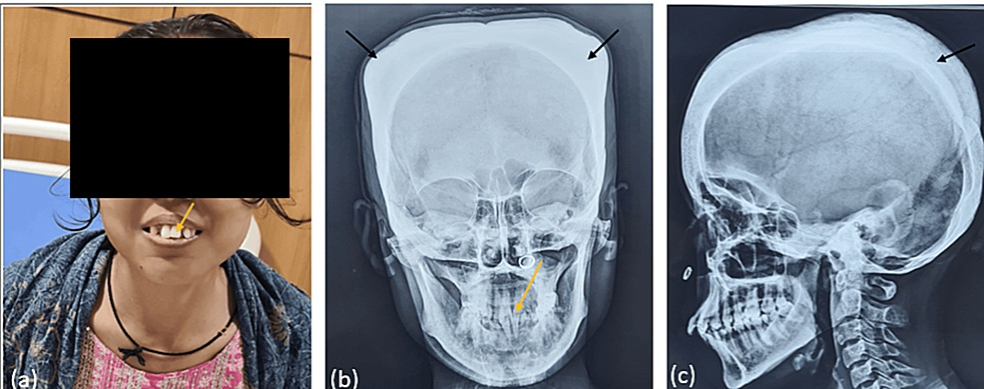
(a, b) Malocclusion of teeth (yellow arrow). (b, c) Parietal bossing (black arrow)

A female aged 30 years came with the chief complaint of weakness, pallor, easy fatiguability, and low hemoglobin (Hb) since childhood. Further inquiry revealed intermittent blood transfusions at ages 16, 17, and 18. Interestingly, she managed to survive at such low Hb levels without requiring regular transfusions until her pregnancy at 27 years old, during which she received 22 units of packed red blood cells, followed by three to four units annually. No history of jaundice, melena, bleeding per rectum, infection, fever, oral ulcer, photosensitivity, tingling, numbness over extremities, or pain involving the chest, abdomen, or joint was reported. Her menstrual history was normal, and she had a normal vaginal delivery without any postpartum complications. No significant family history or consanguinity was reported. The patient was conscious and well oriented during her physical examination. All her vitals were within normal range. Pallor, low body mass index, malocclusion of teeth, frontal/parietal bossing, and hemolytic facies were observed on physical examination (Figure 1). Hepatosplenomegaly was noted, while no lymphadenopathy was observed.

Based on the clinical symptoms and physical examination, the patient was advised to have a complete blood count (CBC), peripheral blood smear (PBS), and reticulocyte count (RC). CBC examination showed TRBC = 1.28 × 106/microliter; Hb = 4.2 gm/dl; hematocrit = 10.8%; mean corpuscular volume (MCV) = 83 fl; mean corpuscular Hb = 29 g/dl; mean corpuscular Hb concentration = 34.9 g/dl; and red cell distribution width = 30.4%. The total leucocyte count was 450/m^3^, and the platelet count was 206 × 10/microliter. On the PBS, normocytic normochromic anemia and anisopoikilocytosis in the form of spherocytes and tear drop cells were seen. nRBC 3/100 WBC were seen. The total leukocyte count was in the normal range (4,500/microliter), and no abnormal cells were seen. The  platelet count was adequate with normal morphology. The RC of the patient was also high, i.e., 6.2%. However, the absolute RC was 1.3%, which was lower for the degree of anemia. On the liver function test, total bilirubin was 1 mg/dL, direct bilirubin was 0.5 mg/dL, serum glutamic oxaloacetic transaminase was 48 U/L, serum glutamate pyruvate transaminase was 57 U/L, and alkaline phosphatase was 110 U/L. The renal function test was essentially within the normal range. Therefore, suspecting this case as that of congenital hemolytic anemia, serum LDH, indirect and direct Coombs test, HPLC, Heinz body test, HbH inclusion test, alpha thalassemia gene analysis, EMA binding test, stool examination for occult blood, serum ferritin, transferrin saturation, and total iron binding capacity tests, along with enzyme studies, were performed (Table 1).

**Table 1:** Laboratory workup of the patient EMA, eosin-5'-maleimide; G6PD, glucose-6 phosphate dehydrogenase; Hb, hemoglobin; HPLC, high-performance liquid chromatography; LDH, lactate dehydrogenase

Parameters	Results	Reference range
Serum LDH	441 U/L	120-246 U/L
Indirect Coombs test	Negative	Negative
Direct Coombs test	Negative	Negative
HPLC		
Hb A	95.9%	92.4-97.6%
Hb A2	2.3%	1.5-3.5%
Hb F	1.1%	0.0-1.0%
Unknown Hb	0.7%	0%
Heinz body test	12%	<35%
HbH inclusion test	Negative	Negative
Alpha thalassemia gene analysis: HbA1 and HbA2	No variation identified	No variation
EMA binding test	91%	88.8-116.7 (MFI: 91.93%)
Stool examination for occult blood	Negative	Negative
Serum ferritin	1,880 ng/ml	10-210 ng/ml
Transferrin saturation	96.08%	20-60%
Total iron binding capacity	252 μg/dl	250-450 μg/dl
Enzymopathies		
Pyruvate kinase	9.0 IU/gHb	5-14 IU/gHb
G6PD	12.0 IU/gHb	9-20 IU/gHb
Glucophosphate isomerase	19.1 IU/gHb	16.3-24.7 IU/gHb

**Figure 2:**
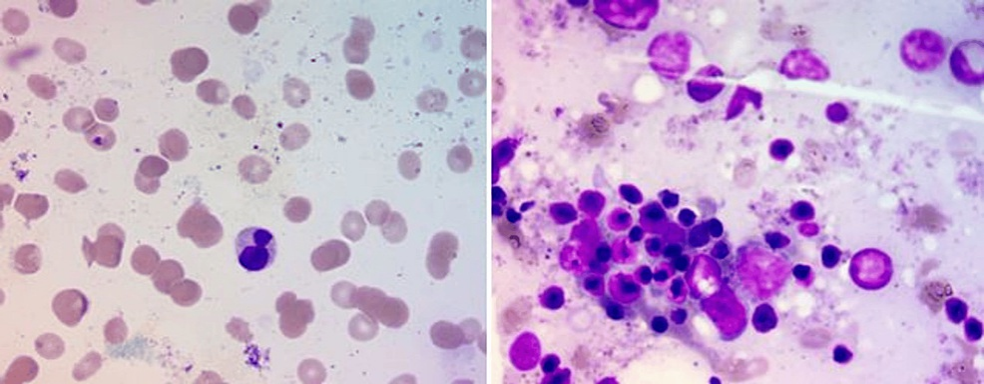
Bone marrow aspirate smears showing (a) binucleated erythroblast and (b) megaloblastic changes (May-Grünwald Giemsa stain: 100x)

**Figure 3:**
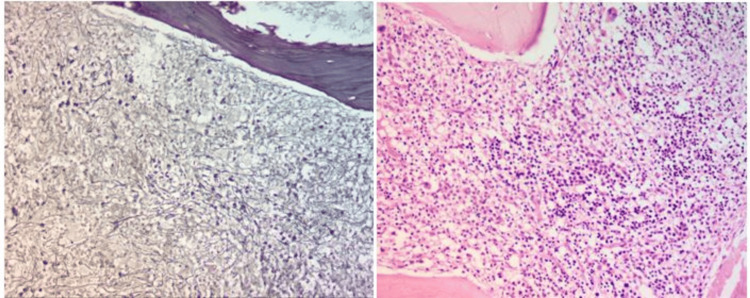
Hypercellular bone marrow biopsy section showing (a) myelofibrosis grade I-II (reticulum stain: 20x) and (b) erythroid hyperplasia (H&E stain: 20x)

Based on the workup, the possibility of having an iron deficiency, enzymopathies, autoimmune hemolytic anemia, or hemoglobinopathies was ruled out, and no specific diagnosis could be made. Therefore, the patient’s consent was obtained, and her blood sample was sent for a hereditary hemolytic anemia gene analysis test, where a total of 48 genes were studied, covering 100% of their coding region. The results showed a homozygous missense mutation in the *SEC23B* gene on chromosome 20 that resulted in the amino acid substitution of cysteine for tyrosine at codon 462. To further complete the hematological workup, a bone marrow aspiration and biopsy were performed. The aspiration smears were partially hemodiluted; however, few hematopoietic elements were seen, and some of the erythroblasts showed megaloblastic changes and dyserythropoiesis in the form of binucleation (Figure 2). The bone marrow biopsy was hypercellular, revealing erythroid hyperplasia, megaloblastic changes, and myelofibrosis grade I-II (WHO 2017), which was quite a unique finding (Figure 3). Both the myeloid and megakaryocytic series were adequate and showed normal morphology.

Hence, the case was diagnosed as CDA type II with myelofibrosis.

## Discussion

CDA type II, also known as hereditary erythroblastic multinuclearity with positive acidified serum lysis test (HEMPAS), is a rare autosomal recessive disorder in which there is a defective assembly of COP II components due to a mutation in the *SEC23B* gene. The mutation in the *SEC23B *gene is detected by homozygous mapping. The COP II components regulate the accumulation of cargo proteins, the deformation of the membrane, and the anterograde transport of correctly folded cargo from the endoplasmic reticulum to the Golgi apparatus. According to a few studies, apart from the *SEC23B *gene mutation, there is a second unidentified mutation in the noncoding regulatory region of the gene that is still unidentified [3]. The majority of CDA type II  cases show missense mutations followed by nonsense, frameshift, splice site,  intronic, and small indel mutations. The compound heterozygosity for missense and nonsense mutations produces more deleterious effects [6]. In our case, although the symptoms started at an early age, the diagnosis was made at the age of 30 years, which is concordant with the study done on 101 CDA type II patients, where the mean age of diagnosis was 37 years [7]. CDA cases have a wide range of heterogeneous clinical presentations, which creates confusion with other congenital hemolytic anemias, most commonly hereditary spherocytosis, and hence results in delayed diagnosis [8]. Such a scenario where the patient managed to survive at extremely low  Hb (4 g/dl) without receiving regular transfusions is quite astonishing.

The presence of clinical manifestations and classic bone marrow findings are the foremost data needed to suspect CDA type II. The bone marrow examination shows erythroid hyperplasia and >10% bi/multinucleated late and intermediate erythroblasts. Sometimes pseudo-Gaucher cells can also be found in the marrow [9]. In this case, the probable reason for repeated transfusions could be attributed to the development of myelofibrosis. For confirmation of this rare diagnosis, we need advanced techniques such as sodium dodecyl sulfate polyacrylamide gel electrophoresis (SDS-PAGE) analysis of red cell membrane proteins and electron microscopy. The electron microscopy shows a double membrane running within the cell membrane of late erythroblast, while on SDS-PAGE analysis, an abnormality in band 3 is seen [10]. Due to financial constraints, these techniques are available in limited laboratories in India, and therefore diagnosis by analyzing molecular defects in the *SEC23B* gene becomes the mainstay for confirmation of CDA type II. The early and accurate diagnosis of CDA type II using molecular diagnostic tools could be immensely useful in initiating stem cell transplantation.

So, in our case, after excluding all the causes of hemolytic anemia, the gene analysis panel was performed, giving us the final diagnosis of CDA type II. The bone marrow examination also showed dyserythropoiesis, which is concordant with CDA type II findings. However, the unusual presentation of myelofibrosis grade 1-II (WHO 2017) on biopsy was unlikely for CDA type II. Due to the delayed diagnosis of CDA type II in this case, the patient was managed on regular blood transfusions only and was doing well until the last follow-up.

## Conclusions

This case report emphasizes the role of molecular genetic testing in solving such clinical quandaries as severe anemias of unknown etiology. These molecular tests play an immense role in early and accurate diagnosis, which in turn could help in appropriate treatment planning (stem cell transplantation) and proper genetic counseling. The prevalence of CDA type II is still unknown owing to its underdiagnosis and underreporting. Therefore, all cases of persistent anemia presenting in childhood should be extensively investigated and reported. Consequently, we can infer that this CDA type II case has a variable clinical presentation and a unique biopsy finding of myelofibrosis, making it worth reporting.

## References

[1] Wolff JA, Von Hofe FH (1951). Familial erythroid multinuclearity. Blood.

[2] Heimpel H, Wendt F (1968). Congenital dyserythropoietic anemia with karyorrhexis and multinuclearity of erythroblasts. Helv Med Acta.

[3] Iolascon A, Heimpel H, Wahlin A, Tamary H (2013). Congenital dyserythropoietic anemias: molecular insights and diagnostic approach. Blood.

[4] Al Hussien HF, Al-Ekeer BN, Serhan HA, Haddadin I, Nashwan AJ (2023). Rare congenital dyserythropoietic anemia of childhood: a case report. Clin Case Rep.

[5] Iolascon A, Russo R, Esposito MR (2010). Molecular analysis of 42 patients with congenital dyserythropoietic anemia type II: new mutations in the SEC23B gene and a search for a genotype-phenotype relationship. Haematologica.

[6] Musri MM, Venturi V, Ferrer-Cortès X (2023). New cases and mutations in SEC23B gene causing congenital dyserythropoietic anemia type II. Int J Mol Sci.

[7] Bianchi P, Schwarz K, Högel J (2016). Analysis of a cohort of 101 CDAII patients: description of 24 new molecular variants and genotype-phenotype correlations. Br J Haematol.

[8] Kedar P, Parmar V, Devendra R, Gupta V, Warang P, Madkaikar M (2017). Congenital dyserythropoietic anemia type II mimicking hereditary spherocytosis in Indian patient with SEC23B-Y462C mutations. Ann Hematol.

[9] Sharma P, Das R, Bansal D, Trehan A (2015). Congenital dyserythropoietic anemia, type II with SEC23B exon 12 c.1385 A → G mutation, and pseudo-Gaucher cells in two siblings. Hematology.

[10] Aydin Koker S, Karapinar TH, Oymak Y, Bianchi P, Fermo E, Gozmen S, Vergin C (2018). Identification of a novel mutation in the SEC23B gene associated with congenital dyserythropoietic anemia type II through the use of next-generation sequencing panel in an undiagnosed case of nonimmune hereditary hemolytic anemia. J Pediatr Hematol Oncol.

